# Informing, Coordinating, and Performing: A Perspective on Functions of Sensorimotor Communication

**DOI:** 10.3389/fnhum.2020.00168

**Published:** 2020-05-26

**Authors:** Cordula Vesper, Vassilis Sevdalis

**Affiliations:** ^1^Department of Linguistics, Cognitive Science and Semiotics, Aarhus University, Aarhus, Denmark; ^2^Interacting Minds Centre, Aarhus University, Aarhus, Denmark; ^3^Department of Public Health, Sport Science, Aarhus University, Aarhus, Denmark

**Keywords:** sensorimotor communication, joint action, nonverbal communication, action prediction, dance, music, sport, aesthetics

## Abstract

Sensorimotor communication is a form of communication instantiated through body movements that are guided by both instrumental, goal-directed intentions and communicative, social intentions. Depending on the social interaction context, sensorimotor communication can serve different functions. This article aims to disentangle three of these functions: (a) an informing function of body movements, to highlight action intentions for an observer; (b) a coordinating function of body movements, to facilitate real-time action prediction in joint action; and (c) a performing function of body movements, to elicit emotional or aesthetic experiences in an audience. We provide examples of research addressing these different functions as well as some influencing factors, relating to individual differences, task characteristics, and situational demands. The article concludes by discussing the benefits of a closer dialog between separate lines of research on sensorimotor communication across different social contexts.

## Introduction

Humans have an intrinsic ability to interact socially with others. From an early age, and before cultivating the language faculty, humans are able to understand others and to be understood by others through pre- and nonverbal cues, such as pointing gestures and gaze direction (Tomasello, 2019). Later in life, this ability becomes particularly relevant in social contexts where the environment prevents verbal exchange (e.g., due to background noise) or where linguistic forms of communication are not appropriate (e.g., in sport and performing art contexts). In such cases, individuals express meaning through their actions and body movements. Nonverbal forms of communication are pertinent in human cultures worldwide (Matsumoto, 2006) and occur in various contexts, from complementing or replacing verbal communication in everyday interactions (Vesper and Richardson, 2014; Peeters et al., 2015; Vesper et al., 2017b; Pezzulo et al., 2019) to supporting complex interpersonal interactions and producing art through dance and music (Sevdalis and Keller, 2011a, 2014; D’Ausilio et al., 2015; MacRitchie et al., 2017; Bishop et al., 2019).

In the past decades, considerable attention has been placed on understanding the foundations of cognitive and social processes within human actions and embodied interactions (Gallese, 2007). Within an embodied cognition framework, bodily movements and sensorimotor experiences are considered pivotal in shaping cognitive functions such as learning, memory, and perception (Wilson, 2002; Barsalou, 2008). One consequence of the embodied nature of cognition is that individuals employ their sensorimotor skills when observing the actions of other individuals (Blakemore and Frith, 2005; Wilson and Knoblich, 2005; Grafton, 2009; Schubert and Semin, 2009). This action simulation or motor resonance is regarded as a fundamental mechanism for social cognition, bridging the gap between self and other (Prinz, 1990; Jeannerod, 2006; Vesper et al., 2010; Herwig et al., 2013). Specifically, the direct matching between action and perception can act as a foundation for the coupling of individual minds and the emergence of sensorimotor communication between them.

In contrast to many predominantly communicative actions such as gesturing while speaking, sensorimotor communication is instantiated through actions that are guided by both communicative, social intentions and by instrumental, goal-directed intentions. To illustrate how this double nature of sensorimotor communication can serve different functions, consider the following example: While continuously playing her musical instrument and producing a desired complex sound pattern (an instrumental action goal), an ensemble musician can inform another performer about her intention to enter a specific musical passage by exaggerating the movement of her upper body (a communicative action goal). A second musician can understand this intention and respond by slowing down the musical tempo so that they play together in synchrony. For an observing audience, the musicians’ coordinated movements can elicit aesthetic experiences and emotional reactions. The musicians’ expertise, their experience with each other’s playing style, and their shared musical and cultural backgrounds can all influence their resulting performance (cf. Keller, 2014).

**Figure 1:**
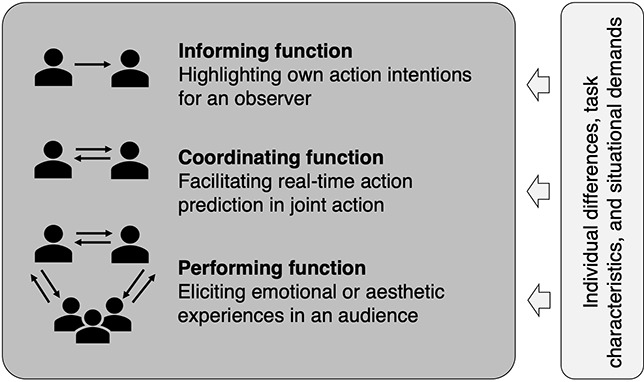
An illustration of three central functions of sensorimotor communication, indicating the main directionality of information exchange between individuals and potential influencing factors on their communication. The number of involved individuals can vary in each context.

A multitude of research studies identified kinematic parameters such as movement amplitude or grasp size that are modified depending on an agent’s action intention (for an overview and discussion, see Ansuini et al., 2014). Previous research on sensorimotor communication examined how such kinematic parameters are modified in joint action to be informative for a co-actor. To that end, a computational model linked movement modifications to internal predictive processes and postulated that sensorimotor communication serves the purpose of facilitating prediction for an observer (Pezzulo et al., 2013). Moreover, a recent framework classified various forms of verbal and nonverbal information exchange (Pezzulo et al., 2019). With this article, we intend to complement such accounts by focusing on the functions that sensorimotor communication serves within different social interaction contexts. In particular, we distinguish three central functions—informing, coordinating, and performing—that differ in the directionality of information flow between individuals, as illustrated in Figure 1.

Accordingly, the section “Informing Function: Highlighting Own Action Intentions for an Observer” introduces movements that provide relevant information to another person, by highlighting an individual’s action intentions. The section “Coordinating Function: Facilitating Real-Time Action Prediction in Joint Action” continues with movements that allow close coordination between multiple individuals’ actions, by facilitating mutual predictions in real-time. The section “Performing Function: Eliciting Emotional or Aesthetic Experiences in an Audience” addresses movements that support performance in contexts such as music and dance, by conveying dynamic expressive nuances that elicit emotional and aesthetical experiences in an audience. These three functions of sensorimotor communication can be influenced by several factors related to individual differences (e.g., in self-report measures of empathy or sensorimotor expertise), specific task characteristics (e.g., when interacting individuals have asymmetric access to task-relevant information), or situational demands (e.g., in a music performance context). To conclude, the section “Final Remarks” discusses the potential benefits of a closer dialog between separate lines of research addressing sensorimotor communication.

## Informing Function: Highlighting Own Action Intentions for an Observer

The first function of sensorimotor communication, that we address in this article, is providing another person with information about one’s intended body movements. It is well established that individual observers are sensitive to information about others’ movement intentions, allowing them to predict what another person will do next (Graf et al., 2007; Becchio et al., 2012; Cavallo et al., 2016). For example, observers can reliably distinguish different social intentions towards another person, such as giving instructions or requesting information (Manera et al., 2010). Factors such as movement complexity, amount of visual information about the movement, and exposure duration to the movement have frequently been related to higher recognition rates (Pollick et al., 2003; Dahl and Friberg, 2007; Sevdalis and Keller, 2009, 2010).

Given this evidence, it could be argued that movements are informative *per se*. In many social contexts, however, movement information is not just passively transmitted as a byproduct of acting; instead, individuals often deliberately modify their movements to make their action intention visible to others. Thus, movements are intentionally modified to be (even more) informative. This function of sensorimotor communication occurs most prominently in situations where making another person aware of one’s intention is explicitly desired, such as during teaching and demonstration. For instance, a dance teacher might exaggerate movement cues to make her pupils understand what is most important to imitate, while she continues to perform the dance movement itself. Empirical research reveals the flexibility with which certain features of an action can be modified to fulfill the informative function of sensorimotor communication. As an example, individuals, who teach an observer a particular musical sequence, exaggerate kinematic features such as amplitude and velocity so that they become informative about where in space the movement is directed towards (McEllin et al., 2018). Similarly, in child-directed action, it has been shown that adults tend to modify movement cues to teach a child, for example, how to use a novel tool (“motionese”; Brand et al., 2002). This sensorimotor communication is thought to support learning by highlighting and separating the relevant action steps (Koterba and Iverson, 2009; Williamson and Brand, 2014). Even minimal modifications are sufficient for supporting the recognition of intentions. For instance, movements produced in a joint action context often contain sufficient information, so that even individuals unrelated to a specific interaction context can predict the actors’ movement goals from simple static images (Vesper and Richardson, 2014) or temporal cues (Vesper et al., 2017b).

Although most research has investigated communicative action modulations in cooperative contexts, they also occur in competitive contexts, where movements are intentionally modified to be less informative or misleading. One domain is competitive sports, where players might encounter deceptive body movements from their opponents. By deliberately providing “fake” information about one’s action intention, players can attempt to disturb an opponent’s prediction processes (Cañal-Bruland, 2017), misleading them about the upcoming action and, therefore, eliciting an inappropriate response. In cases such as handball, rugby, and basketball, expert performers have demonstrated a perceptual advantage in correctly disambiguating others’ movement intentions (Cañal-Bruland and Schmidt, 2009; Sebanz and Shiffrar, 2009; Brault et al., 2012; Mori and Shimada, 2013), highlighting the role of action expertise in sensorimotor communication.

## Coordinating Function: Facilitating Real-Time Action Prediction in Joint Action

Beyond merely providing information, body movements can also support real-time coordination between multiple individuals’ actions. In joint action settings, it is often not only necessary to understand the partner’s immediate action intention, that is, what this person is going to do next, but also to be able to perform an appropriate complementary action at the right time, that is, to choose which respective action to perform and when to act. In other words, the real-time constraints and mutual influences between co-actors place a high burden on joint planning and performance. Sensorimotor communication, here, plays the role of a “coordination smoother” (Vesper et al., 2010), a way of simplifying coordination.

One of the most studied contexts of this coordinative function of sensorimotor communication is that of achieving synchrony between two individuals’ actions. In a study with expert pianists playing duets, it was observed that restricting access to shared auditory information made the players visually enhance their finger movement height, which, in turn, allowed them to maintain precise temporal coordination with each other (Goebl and Palmer, 2009). Sensorimotor communication, in this case, compensated for missing auditory information through another (here: visual) modality. Similar findings were obtained in a joint sequence coordination task, where those persons in a dyad who received prior information about upcoming target locations, deliberately modulated their movement amplitude while interacting (Vesper and Richardson, 2014). In particular, they moved to relatively far targets with a higher amplitude and a different velocity profile than to relatively close locations. This modulation allowed the co-actors, who did not receive prior information, to anticipate the location of the correct target and their partners’ movements more efficiently. The impact of sensorimotor communication on the outcome of a joint action was directly tested in a study that modulated the type of perceptual information shared between co-actors (Vesper et al., 2016). Pairs of participants synchronized the endpoints of simple target-directed movements. Compared to a condition without visual access to each other’s movements, participants exaggerated the amplitude of their movements in a condition with visual access. This in turn, allowed them to be more synchronized. Other action features besides movement amplitude are modulated for communication, including grasp position on an object (Schmitz et al., 2018), grasp aperture while moving towards an object (Sacheli et al., 2013), and the speed with which to approach a target location (Vesper et al., 2017b).

Individual differences in co-actors’ social skills or their interaction roles can influence the execution of body movements. Several studies demonstrated that leader/follower relationships influence how dyads or larger groups approach a task, and how well they manage to adjust their movements and achieve fine temporal and spatial coordination with each other (Konvalinka et al., 2010; Glowinski et al., 2013; Badino et al., 2014; Curioni et al., 2019). The assignment of leadership roles can also alter the duration of gaze towards the co-performer in duetting pianists (e.g., before tempo changes), affecting how well they achieve musical synchronization (Kawase, 2014). Coordination of music and body movement can also be influenced by familiarity with a co-performer’s musical part and previous rehearsals (Williamon and Davidson, 2002; Ragert et al., 2013; Keller, 2014). Similar effects likely extend into movement performance in contexts that foster sensorimotor communication. One study tested the influence of inter-individual differences on the emergence of new communication systems (Volman et al., 2012). Their findings suggest that individuals tend to differ in their ability to understand another person’s movement intentions, which, in turn, can affect the success with which dyads manage to creatively invent non-conventional ways of communicating.

## Performing Function: Eliciting Emotional or Aesthetic Experiences in an Audience

In the interaction contexts described in the previous sections, the focus is on informing or transferring task-relevant information to another individual or completing tasks together. In contrast, many situations, such as dance and music performances, can generate more complex interactions, where performers engage in deliberate modulations of their bodily movements to convey meaning to an audience, such as eliciting emotions, aesthetical experiences, or narratives in an observer’s mind. As illustrated in Figure 1, this transforms the overall interactive structure by adding more roles (i.e., performer and audience) and, possibly, more people (although this does not exclude contexts with only one performer, more than two performers, or varying sizes of the audience).

Performers’ movements are potent carriers of aesthetic significance and often convey spatial and temporal expression dynamics in visual and auditory modalities, which influence the experience of observers (Vines et al., 2006; MacRitchie et al., 2013). For example, musical performances are judged as more interesting when, in addition to hearing the music, observers can see the musicians playing in an expressive compared to an inexpressive manner (Broughton and Stevens, 2009). Similarly, dance movements depicting greater displacement of a dancer’s body in space are associated with higher liking ratings from spectators (Calvo-Merino et al., 2008). In contrast to static movement displays, dynamic displays of movements across time provide audiences with information that allow them to infer information about a performer’s intended artistic expression which, for example, allows them to differentiate between expressive and inexpressive motion cues in performing musicians or dancers (Sevdalis and Keller, 2012; MacRitchie et al., 2017). Moreover, aesthetic responses to dance movements can be intensified by dance performers’ interpersonal synchrony (Vicary et al., 2017). Thus, body movements are effective channels for the communication of performers’ intentions for expression, as well as for inducing aesthetic experiences in an observing audience.

Various factors can influence how accurately others’ intentions about expression intensity and aesthetic significance can be perceived in performance contexts. One important factor is performers’ and observers’ sensorimotor expertise, which can emerge from the long-term cultivation of a sensorimotor skill and deliberate practice. In the field of music, for example, expert pianists, organ players, and orchestral conductors were shown to be able to reliably distinguish whether recordings of music performances involve previously executed actions of themselves or other individuals (Keller et al., 2007; Gingras et al., 2011; Wöllner, 2012a). Another important factor, apart from long-term or domain-specific expertise, is incidental sensorimotor experience with an action or an interaction partner, which can be beneficial in a communication process. When observers were asked to identify the intended expression intensity of non-expert dancers, recognition accuracy differed depending on whether the observers had motor experience (observing their own dancing movements), visual experience (observing movements of a dancing partner), or no experience with the displayed actions (observing the dancing movements of a stranger; Sevdalis and Keller, 2011b). A further example of individual characteristics that may influence sensorimotor communication is trait-like individual differences, for example, related to empathy. Individuals scoring higher on empathy in self-report questionnaires were found to be more accurate in estimating performers’ intentions for expression, whether they were observing ensemble musicians (Wöllner, 2012b) or dancers (Sevdalis and Keller, 2012; Sevdalis and Raab, 2014; Sevdalis and Raab, 2016).

## Final Remarks

The present article illustrates the complexity that research on sensorimotor communication in social interaction needs to address: Studying only simple information exchange may not suffice to fully understand how body movements are used to facilitate coordination between individuals; studying only coordination of body movements in a dyadic setting may not suffice to understand the dynamics of interaction between co-performers’ expressions and an audience’s aesthetic responses; focusing only on complex applied performance contexts may not benefit from basic research that addresses low-level sensorimotor processes. Thus, to investigate such complexity, future research will benefit from a multidisciplinary dialog across fields as varied as human movement science, joint action, communication studies, and performance psychology (Vesper et al., 2010, 2017a; D’Ausilio et al., 2015; Sevdalis and Wöllner, 2016).

To date, despite the considerable potential of crosstalk between different fields that focus on communicative actions, the systematic investigation of movements still receives less attention compared to the study of other cognitive processes (Rosenbaum and Feghhi, 2019). Accordingly, to complement the literature on basic motor processes in sensorimotor communication (e.g., Pezzulo et al., 2013; Vesper and Richardson, 2014; Vesper et al., 2017b), we aim to extend the discussion to studies illustrating how movements serve as carriers of meaning and expression dynamics in performing arts contexts (Sevdalis and Keller, 2011a, 2014; Sevdalis and Wöllner, 2016). We hope our proposed schema in Figure 1 will support the systematic assessment of different parameters in human social interaction, such as individuals’ goals that necessitate certain body movements, the contexts in which the movements are embedded, or the particular characteristics of the individuals executing and perceiving these movements. In particular, future research could directly compare contexts that differ only in the function that sensorimotor communication has, and, thereby, specify which influencing factors are particularly relevant to which context.

In our view, the study of sensorimotor communication can enhance our understanding of human cognitive processes more generally, by offering an interactive approach, where cognitive processes do not lie just in one individual mind, but where an acting individual/performer and a partner/audience are investigated as participatory agents in a large-scale communication process. As individuals possess considerable abilities in providing social information to others through their movements and in inferring social information from the subtle movement cues of others, this eventual attunement to mutually exchanged cues can be regarded as a fundamental characteristic of the sensorimotor basis of human social cognition. Besides, going beyond human social processes, a systematic investigation of different functions of sensorimotor communication also promises to be informative for applied research on artificial agents. Just as when two or more humans work together in proximity, and with high temporal and spatial precision, humans interacting with robots may also benefit from the direct and fast information exchange instantiated through nonverbal communicative cues (Dragan and Srinivasa, 2014; Vesper, 2014; Donnarumma et al., 2018). The future will tell how similar robot behavior needs to be to human behavior, to allow the same smooth and easy interaction that we see when humans play basketball, perform a Bach cantata, or simply shake hands with each other.

## Author Contributions

CV and VS contributed equally to the conceptual development and writing of the article.

## Conflict of Interest

The authors declare that the research was conducted in the absence of any commercial or financial relationships that could be construed as a potential conflict of interest.

## References

[B1] Ansuini C. Cavallo A. Bertone C. Becchio C. (2014). The visible face of intention: why kinematics matters. Front. Psychol. 5:815. 10.3389/fpsyg.2014.00815 25104946 PMC4109428

[B630] Badino L. D’Ausilio A. Glowinski D. Camurri A. Fadiga L. (2014). Sensorimotor communication in professional quartets. Neuropsychologia 55, 98–104. 10.1016/j.neuropsychologia.2013.11.012 24333167

[B2] Barsalou L. W. (2008). Grounded cognition. Annu. Rev. Psychol. 59, 617–645. 10.1146/annurev.psych.59.103006.093639 17705682

[B3] Becchio C. Manera V. Sartori L. Cavallo A. Castiello U. (2012). Grasping intentions: from thought experiments to empirical evidence. Front. Hum. Neurosci. 6:117. 10.3389/fnhum.2012.00117 22557961 PMC3340947

[B4] Bishop L. Cancino-Chacón C. Goebl W. (2019). Moving to communicate, moving to interact: patterns of body motion in musical duo performance. Music Percept. 37, 1–25. 10.1525/mp.2019.37.1.1

[B5] Blakemore S.-J. Frith C. (2005). The role of motor contagion in the prediction of action. Neuropsychologia 43, 260–267. 10.1016/j.neuropsychologia.2004.11.012 15707910

[B6] Brand R. J. Baldwin D. A. Ashburn L. A. (2002). Evidence for ‘motionese’: modifications in mothers’ infant-directed action. Dev. Sci. 5, 72–83. 10.1111/1467-7687.00211

[B7] Brault S. Bideau B. Kulpa R. Craig C. M. (2012). Detecting deception in movement: the case of the side-step in rugby. PLoS One 7:e37494. 10.1371/journal.pone.0037494 22701569 PMC3372470

[B8] Broughton M. Stevens C. (2009). Music, movement and marimba: an investigation of the role of movement and gesture in communicating musical expression to an audience. Psychol. Music 37, 137–153. 10.1177/0305735608094511

[B9] Calvo-Merino B. Jola C. Glaser D. E. Haggard P. (2008). Towards a sensorimotor aesthetics of performing art. Conscious. Cogn. 17, 911–922. 10.1016/j.concog.2007.11.003 18207423

[B10] Cañal-Bruland R. (2017). Deception detection in action: embodied simulation in anti-social human interactions. Front. Psychol. 8:166. 10.3389/fpsyg.2017.00166 28223960 PMC5293753

[B11] Cañal-Bruland R. Schmidt M. (2009). Response bias in judging deceptive movements. Acta Psychol. 130, 235–240. 10.1016/j.actpsy.2008.12.009 19193359

[B12] Cavallo A. Koul A. Ansuini C. Capozzi F. Becchio C. (2016). Decoding intentions from movement kinematics. Sci. Rep. 6:37036. 10.1038/srep37036 27845434 PMC5109236

[B13] Curioni A. Vesper C. Knoblich G. Sebanz N. (2019). Reciprocal information flow and role distribution support joint action coordination. Cognition 187, 21–31. 10.1016/j.cognition.2019.02.006 30797991 PMC6446186

[B14] D’Ausilio A. Novembre G. Fadiga L. Keller P. E. (2015). What can music tell us about social interaction? Trends Cogn. Sci. 19, 111–114. 10.1016/j.tics.2015.01.005 25641075

[B15] Dahl S. Friberg A. (2007). Visual perception of expressiveness in musicians’ body movements. Music Percept. 24, 433–454. 10.1525/mp.2007.24.5.433

[B16] Donnarumma F. Dindo H. Pezzulo G. (2018). Sensorimotor communication for humans and robots: improving interactive skills by sending coordination signals. IEEE Trans. Cogn. Dev. Syst. 10, 903–917. 10.1109/tcds.2017.2756107

[B1700] Dragan A. Srinivasa S. (2014). Integrating human observer inferences into robot motion planning. Auton. Robots 37, 351–368. 10.1007/s10514-014-9408-x

[B18] Gallese V. (2007). Before and below ‘theory of mind’: embodied simulation and the neural correlates of social cognition. Philos. Trans. R. Soc. Lond. B Biol. Sci. 362, 659–669. 10.1098/rstb.2006.2002 17301027 PMC2346524

[B19] Gingras B. Lagrandeur-Ponce T. Giordano B. L. McAdams S. (2011). Perceiving musical individuality: performer identification is dependent on performer expertise and expressiveness, but not on listener expertise. Perception 40, 1206–1220. 10.1068/p6891 22308890

[B3000] Glowinski D. Mancini M. Cowie R. Camurri A. Chiorri C. Doherty C. (2013). The movements made by performers in a skilled quartet: a distinctive pattern, and the function that it serves. Front. Psychol. 4. 10.3389/fpsyg.2013.00841 24312065 PMC3826428

[B20] Goebl W. Palmer C. (2009). Synchronization of timing and motion among performing musicians. Music Percept. 26, 427–438. 10.1525/mp.2009.26.5.427

[B21] Graf M. Reitzner B. Corves C. Casile A. Giese M. Prinz W. (2007). Predicting point-light actions in real-time. NeuroImage 36, T22–T32. 10.1016/j.neuroimage.2007.03.017 17499167

[B22] Grafton S. T. (2009). Embodied cognition and the simulation of action to understand others. Ann. N Y Acad. Sci. 1156, 97–117. 10.1111/j.1749-6632.2009.04425.x 19338505

[B23] Herwig A. Beisert M. Prinz W. (2013). “Action science emerging: introduction and leitmotifs,” in Action Science: Foundations of An Emerging Discipline, eds Prinz W. Beisert M. Herwig A. (Cambridge, MA: MIT Press), 1–33.

[B24] Jeannerod M. (2006). Motor Cognition: What Actions Tell the Self. New York, NY: Oxford University Press.

[B25] Kawase S. (2014). Assignment of leadership role changes performers’ gaze during piano duo performances. Ecol. Psychol. 26, 198–215. 10.1080/10407413.2014.929477

[B26] Keller P. E. (2014). “Ensemble performance: interpersonal alignment of musical expression,” in Expressiveness in Music Performance: Empirical Approaches Across Styles and Cultures, eds Fabian D. Timmers R. Schubert E. (Oxford: Oxford University Press), 260–282.

[B27] Keller P. E. Knoblich G. Repp B. H. (2007). Pianists duet better when they play with themselves: on the possible role of action simulation in synchronization. Conscious. Cogn. 16, 102–111. 10.1016/j.concog.2005.12.004 16466932

[B28] Konvalinka I. Vuust P. Roepstorff A. Frith C. D. (2010). Follow you, follow me: continuous mutual prediction and adaptation in joint tapping. Q. J. Exp. Psychol. 63, 2220–2230. 10.1080/17470218.2010.497843 20694920

[B29] Koterba E. A. Iverson J. M. (2009). Investigating motionese: the effect of infant-directed action on infants’ attention and object exploration. Infant Behav. Dev. 32, 437–444. 10.1016/j.infbeh.2009.07.003 19674793

[B30] MacRitchie J. Buck B. Bailey N. J. (2013). Inferring musical structure through bodily gestures. Music. Sci. 17, 86–108. 10.1177/1029864912467632

[B31] MacRitchie J. Varlet M. Keller P. (2017). “Embodied expression through entrainment and co-representation in musical ensemble performance,” in The Routledge Companion to Embodied Music Interaction, 1st Edn., eds Lesaffre M. Maes P.-J. Leman M. (London: Routledge), 150–159.

[B32] Manera V. Schouten B. Becchio C. Bara B. G. Verfaillie K. (2010). Inferring intentions from biological motion: a stimulus set of point light communicative interactions. Behav. Res. Methods 42, 168–178. 10.3758/brm.42.1.168 20160297

[B33] Matsumoto D. (2006). “Culture and nonverbal behavior,” in Handbook of Nonverbal Communication, eds Manusov V. Patterson M. (Thousand Oaks, CA: Sage), 219–235.

[B34] McEllin L. Knoblich G. Sebanz N. (2018). Distinct kinematic markers of demonstration and joint action coordination? Evidence from virtual xylophone playing. J. Exp. Psychol. Hum. Percept. Perform. 44, 885–897. 10.1037/xhp0000505 29154627

[B35] Mori S. Shimada T. (2013). Expert anticipation from deceptive action. Atten. Percept. Psychophys. 75, 751–770. 10.3758/s13414-013-0435-z 23436250

[B36] Peeters D. Chu M. Holler J. Hagoort P. Özyurek A. (2015). Electrophysiological and kinematic correlates of communicative intent in the planning and production of pointing gestures and speech. J. Cogn. Neurosci. 27, 2352–2368. 10.1162/jocn_a_00865 26284993

[B37] Pezzulo G. Donnarumma F. Dindo H. (2013). Human sensorimotor communication: a theory of signaling in online social interactions. PLoS One 8:e79876. 10.1371/journal.pone.0079876 24278201 PMC3835897

[B38] Pezzulo G. Donnarumma F. Dindo H. D’Ausilio A. Konvalinka I. Castelfranchi C. (2019). The body talks: sensorimotor communication and its brain and kinematic signatures. Phys. Life Rev. 28, 1–21. 10.1016/j.plrev.2018.06.014 30072239

[B39] Pollick F. E. Hill H. Calder A. Paterson H. (2003). Recognising facial expression from spatially and temporally modified movements. Perception 32, 813–826. 10.1068/p3319 12974567

[B40] Prinz W. (1990). “A common coding approach to perception and action,” in Relationships Between Perception and Action: Current Approaches, eds Neumann O. Prinz W. (Berlin: Springer), 167–201.

[B41] Ragert M. Schröder T. Keller P. E. (2013). Knowing too little or too much: the effects of familiarity with a co-performer’s part on interpersonal coordination in musical ensembles. Front. Psychol. 4:368. 10.3389/fpsyg.2013.00368 23805116 PMC3691551

[B42] Rosenbaum D. A. Feghhi I. (2019). The time for action is at hand. Atten. Percept. Psychophys. 81, 2123–2138. 10.3758/s13414-018-01647-7 30617768

[B43] Sacheli L. Tidoni E. Pavone E. Aglioti S. Candidi M. (2013). Kinematics fingerprints of leader and follower role-taking during cooperative joint actions. Exp. Brain Res. 226, 473–486. 10.1007/s00221-013-3459-7 23503771

[B44] Schmitz L. Vesper C. Sebanz N. Knoblich G. (2018). When height carries weight: communicating hidden object properties for joint action. Cogn. Sci. 42, 2021–2059. 10.1111/cogs.12638 29936705 PMC6120543

[B45] Schubert T. W. Semin G. R. (2009). Embodiment as a unifying perspective for psychology. Eur. J. Soc. Psychol. 39, 1135–1141. 10.1002/ejsp.670

[B46] Sebanz N. Shiffrar M. (2009). Bluffing Bodies: inferring intentions from actions. Psychon. Bull. Rev. 16, 170–175. 10.3758/PBR.16.1.170 19145029

[B48] Sevdalis V. Keller P. E. (2009). Self-recognition in the perception of actions performed in synchrony with music. Ann. N Y Acad. Sci. 1169, 499–502. 10.1111/j.1749-6632.2009.04773.x 19673830

[B49] Sevdalis V. Keller P. E. (2010). Cues for self-recognition in point-light displays of actions performed in synchrony with music. Conscious. Cogn. 19, 617–626. 10.1016/j.concog.2010.03.017 20382037

[B50] Sevdalis V. Keller P. E. (2011a). Captured by motion: dance, action understanding, and social cognition. Brain Cogn. 77, 231–236. 10.1016/j.bandc.2011.08.005 21880410

[B51] Sevdalis V. Keller P. E. (2011b). Perceiving performer identity and intended expression intensity in point-light displays of dance. Psychol. Res. 75, 423–434. 10.1007/s00426-010-0312-5 20981438

[B52] Sevdalis V. Keller P. E. (2012). Perceiving bodies in motion: expression intensity, empathy, and experience. Exp. Brain Res. 222, 447–453. 10.1007/s00221-012-3229-y 22941314

[B53] Sevdalis V. Keller P. E. (2014). Know thy sound: perceiving self and other in musical contexts. Acta Psychol. 152, 67–74. 10.1016/j.actpsy.2014.07.002 25113128

[B55] Sevdalis V. Raab M. (2014). Empathy in sports, exercise, and the performing arts. Psychol. Sport Exerc. 15, 173–179. 10.1016/j.psychsport.2013.10.013

[B54] Sevdalis V. Raab M. (2016). Individual differences in athletes’ perception of expressive body movements. Psychol. Sport Exerc. 24, 111–117. 10.1016/j.psychsport.2016.02.001

[B47] Sevdalis V. Wöllner C. (2016). “Capturing motion for enhancing performance: an embodied cognition perspective on sports and the performing arts,” in Performance Psychology, eds Raab M. Lobinger B. Hoffmann S. Pizzera A. Laborde S. (Amsterdam: Academic Press Elsevier), 223–234.

[B56] Tomasello M. (2019). Becoming Human: A Theory of Ontogeny. Cambridge, MA: Harvard University Press.

[B57] Vesper C. (2014). How to support action prediction: Evidence from human coordination tasks. The 23rd IEEE International Symposium on Robot and Human Interactive Communication, Edinburgh, 655–659.

[B59] Vesper C. Abramova E. Bütepage J. Ciardo F. Crossey B. Effenberg A.. (2017a). Joint action: mental representations, shared information and general mechanisms for coordinating with others. Front. Psychol. 7, 2039–2039. 10.3389/fpsyg.2016.02039 28101077 PMC5209366

[B61] Vesper C. Schmitz L. Knoblich G. (2017b). Modulating action duration to establish non-conventional communication. J. Exp. Psychol. Gen. 164, 1722–1737. 10.1037/xge0000379 29251985

[B60] Vesper C. Butterfill S. Knoblich G. Sebanz N. (2010). A minimal architecture for joint action. Neural Netw. 23, 998–1003. 10.1016/j.neunet.2010.06.002 20598504

[B58] Vesper C. Richardson M. J. (2014). Strategic communication and behavioral coupling in asymmetric joint action. Exp. Brain Res. 232, 2945–2956. 10.1007/s00221-014-3982-1 24838557 PMC4381276

[B62] Vesper C. Schmitz L. Safra L. Sebanz N. Knoblich G. (2016). The role of shared visual information for joint action coordination. Cognition 153, 118–123. 10.1016/j.cognition.2016.05.002 27183398 PMC4918098

[B63] Vicary S. Sperling M. von Zimmermann J. Richardson D. C. Orgs G. (2017). Joint action aesthetics. PLoS One 12:e0180101. 10.1371/journal.pone.0180101 28742849 PMC5526561

[B64] Vines B. W. Krumhansl C. L. Wanderley M. M. Levitin D. J. (2006). Cross-modal interactions in the perception of musical performance. Cognition 101, 80–113. 10.1016/j.cognition.2005.09.003 16289067

[B65] Volman I. Noordzij M. L. Toni I. (2012). Sources of variability in human communicative skills. Front. Hum. Neurosci. 6:310. 10.3389/fnhum.2012.00310 23189048 PMC3504324

[B67] Williamson R. A. Brand R. J. (2014). Child-directed action promotes 2-year-olds’ imitation. J. Exp. Child Psychol. 118, 119–126. 10.1016/j.jecp.2013.08.005 24079611

[B66] Williamon A. Davidson J. W. (2002). Exploring co-performer communication. Music. Sci. 6, 53–72. 10.1177/102986490200600103

[B68] Wilson M. (2002). Six views of embodied cognition. Psychon. Bull. Rev. 9, 625–636. 10.3758/bf03196322 12613670

[B69] Wilson M. Knoblich G. (2005). The case for motor involvement in perceiving conspecifics. Psychol. Bull. 131, 460–473. 10.1037/0033-2909.131.3.460 15869341

[B70] Wöllner C. (2012a). Self-recognition of highly skilled actions: a study of orchestral conductors. Conscious. Cogn. 21, 1311–1321. 10.1016/j.concog.2012.06.006 22795864

[B71] Wöllner C. (2012b). Is empathy related to the perception of emotional expression in music? A multimodal time-series analysis. Psychol. Aesthet. Creat. Arts 6, 214–223. 10.1037/a0027392

